# Editorial: Near-infrared fluorescence probes for biomedical applications

**DOI:** 10.3389/fbioe.2023.1267302

**Published:** 2023-08-30

**Authors:** Lu Liu, Benhao Li, Peiyuan Wang

**Affiliations:** ^1^ School of Pharmacy, Shandong Technology Innovation Center of Molecular Targeting and Intelligent Diagnosis and Treatment, Binzhou Medical University, Yantai, China; ^2^ Departments of Diagnostic Radiology, Chemical and Biomolecular Engineering and Biomedical Engineering, Yong Loo Lin School of Medicine, National University of Singapore, Singapore, Singapore; ^3^ Key Laboratory of Design and Assembly of Functional Nanostructures, Fujian Institute of Research on the Structure of Matter, Chinese Academy of Sciences, Fuzhou, China

**Keywords:** near-infrared imaging, bioimaging and biosensing, fluorescence imaging, NIR probes, image-guided therapy, image-guided surgery

Near-infrared fluorescence (NIR, 700–1700 nm) imaging offers a promising solution to enhance bioimaging and biosensing in both fundamental research and clinical applications. Its non-invasive nature, deep tissue penetration, high sensitivity, real-time imaging capabilities, and remarkable signal-to-background ratio set it apart as a superior alternative to conventional imaging modalities. Recent advancements in NIR fluorescence imaging have sparked the emergence of novel devices, methodologies, and applications in the biological realm. These include novel NIR materials, improved NIR fluorescence, integration with multi-modality or multi-dimensional approaches, and synergistic combinations with state-of-the-art instrumentation and systems. As a result, NIR fluorescence imaging has emerged as a superior choice for researchers and clinicians seeking efficient and cutting-edge imaging techniques.

The advancement of NIR bioimaging and therapy critically hinges on the artful design, adept synthesis, and skillful engineering of a diverse array of NIR fluorescence probes, with a notable emphasis on pioneering materials and molecules. This Research Topic seeks to highlight the progress made in fundamental science regarding a lot of innovative NIR fluorescence probes, as well as the development of cutting-edge instrumentation and systems. Additionally, the exploration of multi-dimensional imaging approaches and the application of NIR fluorescence in various fields are encouraged to push the boundaries and broaden the horizons of this field.

The continuous progress in the development of cutting-edge NIR fluorescence instrumentation and systems holds great promise in enhancing the spatiotemporal resolution and noninvasive nature of *in vivo* bioimaging, biosensing, and therapy across various disciplines. The early assessment of local tissue oxygen saturation emerges as a critical tool for clinicians to accurately ascertain the severity of burn wounds. Kim et al. assessed the burn extent and depth in the skin of the extremities using a custom-built 36-channel functional near-infrared spectroscopy system in patients with burns. All second-degree burns were classified as shallow, moderate and deep burns. Functional near-infrared spectroscopy showed a significant difference between non-burned skin on the burned side and healthy skin on the contralateral non-burned side. Hemodynamic measurements using functional near-infrared spectroscopy were more consistent with the diagnosis of burns 1 week later than that of the degree of burns diagnosed visually at the time of admission. Therefore, functional near-infrared spectroscopy may help with the early judgment of burn extent and depth by reflecting differences in the oxygen saturation levels in the skin.

In the realm of microsurgery, one perennial challenge lies in precisely identifying the blood supply during intricate procedures like vascular anastomosis, digit replantation, skin avulsion reconstruction, and flap transplantation. Another article by Wu et al. used the portable NIR-II (1,000–1,700 nm) imaging instrument for imaging of 39 patients undergoing microvascular anastomosis, digit replantation, avulsion injury or perforator-based island flap surgery. The patients were injected with indocyanine green (ICG), which can be used for both NIR-I (700–900 nm) and NIR-II imaging and were approved by the Food and Drug Administration (FDA) for routine clinical use. Moreover, the imaging performance of the NIR-Ι window was compared with that of the NIR-ΙI window in each patient, to evaluate the potential of NIR-II image-guided microsurgery in clinic. The study indicates that NIR-II fluorescence imaging with ICG provides a non-invasive, high-resolution, deep penetration, large scale and real-time detection of the cutaneous microvasculature, which makes it a promising tool for the wide spectra of microsurgeries.

NIR fluorescence imaging-guided surgery offers valuable intraoperative image guidance, enabling surgeons to assess surgical margins and detect residual lesions and small metastases with enhanced precision. Nonetheless, the potential of using NIR fluorescence imaging, particularly with methylene blue (MB), for intraoperative navigation of the stomach and gastric tumors requires further investigation and exploration. Wang et al. used MB for the NIR imaging of gastric cancer cell xenografts, suggesting that MB cannot specifically target subcutaneous and orthotopic gastric tumors in xenograft models. NIR fluorophores MB can exhibit specifically uptake by the gastric epithelial and cancer cells.

The immune checkpoint blockade has revolutionized cancer therapy, presenting a groundbreaking approach. Despite this significant advancement, accurately predicting the efficacy of immunotherapy based on PD-L1 expression still poses challenges. Another study by Wei et al. created PD-L1 aptamer-anchored (Hf@ICG-Apt) spherical nucleic acids with a shell made of PD-L1 aptamer and indocyanine green embedded in a mesoporous hafnium oxide nanoparticle core. The nanoprobe enhanced the stability of ICG in aqueous solution, protected aptamer PD-L1 from nuclease degradation, and improved their accumulation in the high PD-L1 expressed tumor sites. Hf@ICG-Apt could figure out the PD-L1 expression differences with NIR-II imaging both *in vivo* and *in vitro*.

Recent advancements in microscopy techniques, coupled with the utilization of near-infrared region fluorophores, have paved the way for exciting opportunities *in vivo* bioimaging. The continual evolution of chemical materials and physical optoelectronics has given rise to the emergence of novel NIR-II microscopy techniques. Among these, confocal and multiphoton microscopy, light-sheet fluorescence microscopy (LSFM), and wide-field microscopy stand out as promising avenues for further progress. These cutting-edge approaches hold immense potential in pushing the boundaries of bioimaging and expanding our understanding of biological processes *in vivo*. Wang et al. highlight the recent advances in the characteristics of *in vivo* imaging using NIR-II fluorescence microscopy and NIR-II fluorescence microscopy techniques in bioimaging and the potential for overcoming current challenges. They emphasize the future challenges and outlooks, optimization of the imaging optical path and the innovation of fluorescence probes will greatly promote the improvement of fluorescence microscopy imaging. The continuous improvements in imaging depth and signal to noise ratio of *in vivo* imaging will drive the vigorous development of NIR-II fluorescence microscopy-based *in vivo* imaging systems and techniques.

